# Development of Evidence‐Based Guidelines for the Integration of Generative AI in University Education Through a Multidisciplinary, Consensus‐Based Approach

**DOI:** 10.1111/eje.13069

**Published:** 2025-02-13

**Authors:** Loizos Symeou, Loucas Louca, Argyro Kavadella, James Mackay, Yianna Danidou, Violetta Raffay

**Affiliations:** ^1^ European University Cyprus Nicosia Cyprus

**Keywords:** AI, artificial intelligence, ChatGPT, consensus, guidelines, higher education, large language models, university education

## Abstract

**Introduction:**

The introduction highlights the transformative impact of generative artificial intelligence (GenAI) on higher education (HE), emphasising its potential to enhance student learning and instructor efficiency while also addressing significant challenges such as accuracy, privacy, and ethical concerns. By exploring the benefits and risks of AI integration, the introduction underscores the urgent need for evidence‐based, inclusive, and adaptable frameworks to guide universities in leveraging GenAI responsibly and effectively in academic environments.

**Aims:**

This paper presents a comprehensive process for developing cross‐disciplinary and consensus‐based guidelines, based on the latest evidence for the integration of GenAI at European University Cyprus (EUC). In response to the rapid adoption of AI tools such as LLMs in HE, a task group at EUC created a structured framework to guide the ethical and effective use of GenAI in academia, one that was intended to be flexible enough to incorporate new developments and not infringe on instructors' academic freedoms, while also addressing ethical and practical concerns.

**Results:**

The framework development was informed by extensive literature reviews and consultations. Key pillars of the framework include: addressing the risks and opportunities presented by GenAI; promoting transparent communication; ensuring responsible use by students and educators; safeguarding academic integrity. The guidelines emphasise the balance between, on the one hand, leveraging AI to enhance educational experiences, and, on the other maintaining critical thinking and originality. The framework also includes practical recommendations for AI usage, classroom integration, and policy formulation, ensuring that AI augments rather than replaces human judgement in educational settings.

**Conclusions:**

The iterative development process, including the use of GenAI tools for refining the guidelines, illustrates a hands‐on approach to AI adoption in HE, and the resulting guidelines may serve as a model for other higher education institutions (HEIs) aiming to integrate AI tools while upholding educational quality and ethical standards.

## 1 Introduction

The introduction of information technology in higher education (HE) offers added value to traditional education and requires higher education institutions (HEIs) to support its academic staff and students to effectively use the technology in a strategic and quality‐based manner [1]. If these principles held true for the introduction of the internet, Web 2.0, e‐learning, and other technological advancements (e.g., virtual reality, simulations) of the last two decades, they are even more pertinent nowadays as Artificial Intelligence (AI) is deployed in HE.

The integration of AI in education, particularly in HE, has ushered in a transformative period for both student learning and academic teaching, impacting all disciplines. Large Language Models (LLMs), such as ChatGPT (Open AI), Claude (Anthropic) and Gemini (Google AI), belong to an AI category known as Generative Artificial Intelligence (GenAI). They are capable of processing user prompts (inputs) in natural language and generating coherent, human‐like text (output). LLMs are trained using large datasets that combine text taken from the internet with other published materials, using machine learning methods, fine‐tuning and self‐attention mechanisms, and they have also been given web search capabilities. This training permits them to recognise the patterns, language, and context of input text: thus, they can be valuable assistants in various fields including research and education [2, 3].

Using LLMs such as ChatGPT in HE has the potential to create a dynamic learning environment and increase the quality of students' learning, as well as instructors' teaching experience [4]. Students experiencing LLM‐enhanced education might be given a highly interactive and customised tutoring environment, receive immediate feedback, and have their queries instantly explained, all of which may improve their academic performance [4]. Improvement in academic performance was evident in an experimental, real‐life implementation of AI, where for example, dental students who developed learning assignments using ChatGPT performed significantly better in the knowledge test than their peers who used the internet search engines to compile their assignments [5]. Similarly, it has been concluded that integrating ChatGPT in online learning environments increased students' self‐regulation levels, confidence and satisfaction [6]. Instructors utilising LLMs could delegate routine administrative tasks and thus focus on course design and innovative pedagogical methods [7, 8]. Therefore, they may enhance their teaching (by creating learning materials, quizzes, summaries, and by teaching students how to critically evaluate information), as well as their assessment methods (by designing reflective assessment activities and innovative assessments promoting students' creativity, as well as rethinking evaluation criteria) [9]. However, concerns about the educational effectiveness of LLMs are also highlighted in recent literature: for instance, an experimental study which investigated the use of ChatGPT effects on university students' creative writing abilities reported a significant decline in students' creative writing as a result of using the chatbot [10]. Drawing evidence‐based conclusions about AI's educational effectiveness is challenging, because of the lack of robust evidence of implementing AI technologies in education [11] and the limited amount of high‐quality, experimental research [12].

Despite the potential advantages and possibilities of GenAI in education, there are limitations and concerns associated with its use: information accuracy and hallucinations, user data privacy, lack of transparency of data sources, bias, discrimination, and ethical issues, including plagiarism and academic dishonesty [13]. In an (unsuccessful) effort to address these issues, some higher education institutes (HEIs) banned the use of GenAI entirely, characterising it as cheating [7, 14, 15]. For example, Hong Kong University initially banned the use of ChatGPT for credit‐bearing assignments, considering its use as plagiarism. However, the same university embraced the technology 6 months later, implementing a new policy wherein students and instructors had free access to ChatGPT and image generator Dall‐E [16]. As GenAI becomes more embedded in all areas of academic life, with both instructors and learners asking for guidance, it is imperative that universities develop and implement clear guidelines and policies on its responsible use [14, 17]. Such clear policies will enable HEIs to harness GenAI's benefits while addressing ethical considerations and ensuring learning outcomes.

A 2023 review of the world's 50 top‐ranking HEIs concluded that universities should progressively adopt the use of AI and develop guidelines to regulate its ethical and efficient implementation [14]. However, at the time, less than half of these universities had developed such guidelines, and those guidelines which had been developed mainly covered the areas of academic integrity, assessment design and communication with students [14]. Similarly, an analysis of institutional guidelines of a number of universities indicated that, although 73% of institutions encouraged the use of GenAI, only 48% provided specific guidance on how to do so [18]. Another recent survey of the adoption strategies of 40 universities across six global regions explored how new technologies such as AI are integrated within the complex systems of HEIs [19]. Specifically, the study investigated characteristics of GenAI innovation including compatibility, trialability and observability, examining the use of communication channels and the policies describing the roles and responsibilities of stakeholders. The study concluded that although universities attempt to actively integrate AI with a focus on academic integrity, teaching, and cultivation of 21st‐century skills, they are still unprepared to fully leverage AI innovations [19]. The study also identified deficiencies in comprehensive policy development, communication channels, and available resources; authors emphasised that collaborative efforts and active involvement of all stakeholders are necessary for successful AI adoption.

To address the integration of AI in university teaching and learning, Chan [20] proposed an AI Policy Education Framework, organised into the dimensions of pedagogy, governance and operations. Building on Chan's Framework and using feedback from academics and students, Cacho [21] developed specific guidelines across six sections, namely: the rationale for AI implementation; the Institution's position on AI tools; key terms; guidelines for teachers; guidelines for students; guidepost. These flexible guidelines can be adopted and modified by institutions and individuals, tailored to their specific visions and needs.

Commonalities in all frameworks and guidelines on AI inclusion in HE include: the Institution's vision and goals; the infrastructure/resources and operational challenges; the stakeholders' involvement (faculty, students and administration); the structured methodology; the provision of guidelines for students and instructors; the communication channels; and the training on AI of students and instructors (AI literacy). The latter has emerged as an urgent issue accompanying the implementation of GenAI in education: training opportunities for both students and faculty are increasingly recognised as essential to ensure effective and responsible integration into academic practises, addressing both technical competencies and ethical considerations [3, 4, 7, 14, 19, 20, 22]. To tackle this challenge, Schwendicke et al. [23] proposed a core curriculum for dental education, aiming to increase educators' and learners' AI literacy and allow them to critically appraise and responsibly use AI applications. The learning outcomes of this curriculum include the domains of basic knowledge, use cases, evaluation, and governance, and accountability, and can be more widely utilised in HE, extending beyond the dental field.

To our knowledge, although guidelines and frameworks for GenAI deployment in HE have been proposed and researched, there is no study as yet reporting on the actual development process of such guidelines, organised at the university level. Therefore, in this study, we describe the process for the development of evidence‐based, multidisciplinary, and consensus‐based guidelines for efficient and ethical integration of AI at the University. These guidelines were developed by a dedicated task group within EUC, comprised of faculty members of different disciplines alongside IT experts and an instructional designer. They were informed by a literature search, relevant documents from international agencies [11, 24] and educational organisations [25], and experts' webinars on the implementation of AI by HEIs [26, 27]. The framework was produced through a consensus process among the task group members, and then reviewed and complemented by the GDPR officer and the Specialist on Inclusive Education, Assistive Technology, and Accessibility. In the end, the amended document was refined using GenAI after training the model with examples of specific language, and edited by humans. Finally, it was peer‐reviewed by faculty members who had not participated in the task group, and also by students. The Framework is intended for the entire University community, including all Departments and Schools, with a focus on instructors and students. The Framework establishes a broad foundation, leaving a role for Departments in developing for subject‐specific guidelines, as the implementation process continues to unfold. This process, as well as the Framework, may serve as an example of practise for HEIs in their effort to integrate AI tools into education while maintaining the academic integrity and quality of the education provided in their institution.

## 2 Aim

The aim of this study is to describe the process for the development of an evidence‐based, multidisciplinary, and consensus‐based Framework for efficient and ethical integration of GenAI in EUC.

## 3 Methodology

### 3.1 The Context

EUC is located in the capital of an EU member and has around 11 000 students. It serves a varied population, which accounts for around 40% local students, 45% students from the other member states and another 15% other international students. Internationalisation is central to EUC's sustainability alongside with key drivers aiming its sustainable growth, including academic excellence, research innovation and creativity, as well as its engagement with industry and the employability of its graduates. EUC has introduced state‐of‐the‐art technology and boosted the country's health sector through the establishment of the most advanced medical sciences facilities in the region. With its medical science‐based degrees, specifically Medicine, Veterinary Medicine and the first Dentistry programme in the country, speared in four out of its 7 Schools, the University offers a comprehensive selection of studies in the fields of the broader Health Sciences, and attracts academics and students from around the globe. The University is a founding member of a European Universities' Alliance of 9 universities in total funded by the European Commission.

EUC has a strong focus on pedagogy, encompassing extensive processes, infrastructure, and initiatives dedicated to enhancing teaching and learning practises. A major part of EUC's strategic planning focuses on educating and empowering students and academics for successful and fulfilling careers. As part of the implementation of this educational mission, the Office of the Vice Rector of Academic Affairs at EUC runs a 35‐h induction professional development programme on an annual basis for all newly hired academic staff. It aims at the pedagogical training of academics, acknowledging gaps identified by the European Commission [24] in HE instructors' training. The content of the programme focuses on various aspects of teaching and learning in HE. As of the academic year 2021–22, 25 of these hours are provided through the ‘New to Teaching Programme’ of Advance HE, which aims to nurture and develop contemporary professional learning. This programme particularly focuses on effective teaching practise, incorporating the latest innovations in online pedagogies and digital delivery within both fully virtual and hybrid teaching spaces. It models approaches and activities in teaching, positioning the participants as a learner in online spaces and inviting them to reflect critically on the experience in order to enhance their own teaching practises. Upon completion of the programme, participants are granted a certificate of attendance and participation.

At the same time, the University also organises an ongoing professional development programme on an annual basis for both full‐time and part‐time academic staff in continuous employment. The content and topics for the programme are decided based on the feedback and evaluation of the EUC Faculty Professional Development Programme and requests of contemporary issues and initiatives from the academic Schools and Departments. Following current trends these topics include, but are not limited to, pedagogies in HE with a special focus on e‐learning and digital technologies [28], inclusive pedagogies [29], contemporary blended learning approaches [30, 31], online teaching and learning [32] and synergetic learning communities [33, 34].

As part of this educational mission, the Committee on Internal Quality Assurance (C.I.Q.A.) of the University, which runs under the Office of the Vice Rector of Academic Affairs, embarked on a comprehensive initiative to look at ways of integrating GenAI into the educational framework provision. This initiative included different stakeholders that were actively involved in the decision‐making process. The legacy of EUC's response to the COVID‐19 lockdown was a cornerstone of the processes involved. Faced at the time with diverse challenges, EUC swiftly transitioned to online learning for conventional degrees, using robust internal educational and quality assurance processes to ensure that students continued to receive high‐quality education with minimal interruption. This rapid adaptation was made possible by strong internal communication channels, hard work by staff and faculty, and a culture of innovation that permeates the institution [35].

### 3.2 The Process

Frameworks in HE—at institutional, European or broader international level—are frequently developed by ad‐hoc task groups. The task is usually accomplished through a consensus or peer‐reviewed process, which may be informed by literature reviews, questionnaires, surveys, interviews, working groups' deliverables, relevant documentation or other reliable sources [36, 37, 38]. The stated intention is to produce recommendations that are evidence‐based, current, clear and applicable, in order for them to be widely accepted and implemented. For example, the consensus ‘Recommendations for the development of e‐modules for the continuing professional development of European dentists’ were informed by a literature search, consultations from e‐learning and IT experts, discussions among the participants of a special interest group, and feedback from the evaluation of an exemplar e‐module [36]; this is similar to the ‘Guidelines for the organization of continuing professional development activities’, where a literature review, a survey, and discussions were used [37]. Along the same lines, the ‘Spanish curriculum in cariology for undergraduate dental students’ was agreed through a consensus process among 38 experts from 16 universities [38], whereas consensus recommendations for incident learning in radiation oncology were developed by a panel of experts that included representatives from relevant organisations [39]. The majority of GenAI guidelines issued by universities were issued either by the Centre for Teaching and Learning of the University or by the Office of the Provost, as observed in a recent study [14]. In contrast to this approach, EUC opted for a wider, more inclusive approach, where all relevant stakeholders provided feedback on a draft framework developed by the dedicated task group. As a result, the final document emerged as a co‐creation effort, incorporating the collective input and perspectives of all stakeholders involved. The task group served as a central link between educators, specialists, students, university committees, the Vice Rector of Academic Affairs, and the Senate.

The early steps of EUC's preparation towards integrating GenAI into the education framework began with an initial meeting of the ad‐hoc committee on generative AI set up by CIQA in January 2023. This set the stage for subsequent initiatives and discussions. On February 20, 2023, EUC hosted an online round table session titled ‘The Use of Artificial Intelligence (AI) in Higher Education’ as part of the Faculty Professional Development Programme described above. All faculty members were invited to participate in this virtual session, which provided a platform for discussing the potential benefits and challenges of AI in HE.

Then, in July 2023, EUC developed a video module for its academic staff and student body using its in‐house studio, under its ‘Content Factory’ initiative, on the topic of ‘Why ChatGPT ISN'T Plagiarism?’ This module, which concerns the ethical use of GenAI tools in academic work, went live in November 2023 and is available to all students and academic staff of the University. Additionally, from November 2023 to July 2024, EUC facilitated a professional learning community (PLC) on AI. This involved a series of interdisciplinary meetings and knowledge exchanges, fostering collaborative learning among faculty members from various disciplines.

Further to the above, a faculty member was appointed to represent EUC in the ‘European University Association Task‐and‐Finish Group on Artificial Intelligence’. This group, which held its inaugural meeting in March 2024, is dedicated to exploring the implications and applications of AI within the context of HE. It prepares webinars, publishes expert voices articles, and develops best practise documents, including use case examples, to guide universities across Europe in their AI integration efforts. EUC's representative co‐presented a webinar titled ‘Artificial Intelligence and Ethics: The Place of New Technologies within University Missions’ on June 18, 2024. This webinar, which is now available online, discussed the ethical considerations and potential benefits of incorporating AI into university missions and also addressed potential ethical dilemmas arising from this development [27].

**FIGURE 1:**
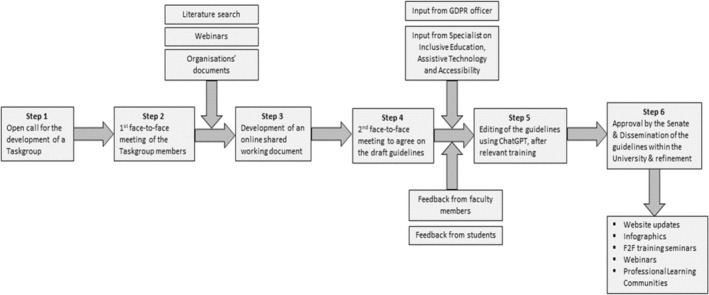
Steps towards the development of the framework on GenAI.

The flowchart illustrated in Figure 1 outlines the systematic methodology followed by EUC for the development and dissemination of a framework including guidelines and suggestions for GenAI integration within the University. The process is outlined in 6 distinct steps, ensuring a structured and collaborative approach. The entire process was concluded in 10 months. Throughout this duration, workshops and webinars were organised for the instructors and students, aiming at familiarising them with the possibilities and limitations of GenAI applications.

The initial step involved the Office of the Vice Rector of Academic Affairs issuing an open call to all faculty members interested in participating in the dedicated AI task group. This call invited faculty across disciplines to contribute to the development of the framework, in order to ensure a broad representation of expertise and perspectives. The task group formed was multi‐disciplinary, reflecting the different needs and particularities of each scientific field in relation to the utilisation of GenAI in HE. Participant faculty included representatives from all academic schools of the University at the time: the School of Medicine; the School of Dentistry; the School of Sciences; the School of Law; the School of Humanities, Social and Education Sciences; the School of Business Administration. An IT expert was also seconded to the task group to provide technical insights into AI implementation, as well as an instructional designer. At the same time, the coordinator of the Digital enhanced Learning and the Professional Development ad hoc Senate Committees of the University also participated in this effort.

Following the formation of the task group, the first face‐to‐face meeting was conducted, whilst, after the initial meeting, the task group collaborated on an online shared working document that served as a central repository for contributions, suggestions, and edits from all members, facilitating continuous development and refinement and ensuring that all members could contribute equally. The document thus evolved with input and insights from diverse disciplines.

The task group then convened for additional meetings, to review the working document and to reach a consensus on the final version of the framework. These meetings were crucial for addressing any remaining issues and ensuring all perspectives are considered, leading to a comprehensive set of usable principles that meet the needs of all disciplines involved.

Once the draft framework was agreed upon, it was shared with the GDPR Officer of the University and an EUC faculty Specialist on Inclusive Education, Assistive Technology, and Accessibility, both of whom provided focused feedback on the sections of the document related to their expertise. Afterwards, feedback on the amended document was solicited from students' elected representatives of all Departments and Schools, and from faculty members who reviewed the document during a face‐to‐face Workshop entitled ‘Culture and Practice for the use of AI’. This 2‐day event aimed to support participants to develop their use of Generative AI to both enhance student learning and also prepare graduates for working with GenAI. Through guided discussion, faculty members considered the potential of AI to change the reality of university communities and explored both possibilities and challenges.

The stakeholders contributing to the development of the framework are in line with the recommendations of Gimpel et al. [9], who proposed that all faculties and disciplines must be included in the process, along with computer scientists, students, didactics experts, legal experts and the IT department.

The final version was edited using ChatGPT 4o, showcasing practical applications of AI in refining the guidelines and demonstrating a hands‐on approach to AI integration. The chatbot was prompted to standardise the framework and stylistically match it with existing EUC policies. The task group members had already received relevant training to effectively utilise this GenAI tool, which has ensured that each point is clear, concise and comprehensive.

The last step involved effective dissemination of the framework throughout the University to make all faculty, staff, and students aware of its existence and approval. Dissemination activities were enhanced through training events including workshops and webinars, as well as Professional Learning Communities. For example, a new Faculty Learning Community was launched, focusing on enhancing the quality of essay‐type assignments' feedback provided to students, using GenAI. The further aim is for staff to develop more subject‐specific advice within their own departments and schools, while adhering to an overall framework that has been adopted across the institution.

In summarising, the characteristics of this process were: (i) it was multidisciplinary, consensus‐based, and inclusive; (ii) a task group acted as a hub among stakeholders; (iii) input was taken from specialists to address specific sections of the framework; (iv) training activities were organised both during the development process and also after it was concluded; (v) the framework was designed to act as umbrella guidance, offering general directions rather than specific instructions; (vi) the framework was conceived as a continuous process, further to be tailored to the requirements of specific disciplines, schools and departments during implementation.

### 3.3 Results and Discussion

The Task Group agreed on a framework for ethical and efficient use of AI in the University, including 10 areas: (1) Purpose and Objectives; (2) Human centricity, Inclusion, Non‐discrimination and Fairness; (3) Respect for human rights and data privacy; (4) Safety and Security; (5) Risks and Opportunities of GenAI; (6) Communication and Transparency; (7) Use of AI by students; (8) Use of AI by educators; (9) AI and GDPR Compliance; (10) Conclusions. During the discussions, examples of good and poor practise regarding the implementation of GenAI were identified and added to the document.

Below, we discuss each of these sections separately, pointing to some of the major debates and or concerns that animated these discussions, with reference to the wider literature that was consulted or drawn on by various committee members.

1. Purpose and Objectives

In writing this section the task group was concerned with explaining the need for a framework, and what it was intended to achieve, as presented below.

**FRAMEWORK OF EUC FOR THE USE OF GENAI FOR TEACHING AND LEARNING**

1. **PURPOSE AND OBJECTIVES**
  1.1 **Purpose**
    1.1.1 The purpose of this framework is to integrate AI tools responsibly within the educational environment while upholding academic integrity, providing guidelines and support for the ethical use of AI by students and educators.
  1.2 **Objectives**
    1.2.1 To provide guidance on how to implement structured GenAI learning approaches for students and academic staff.
    1.2.2 To establish clear guidelines for the appropriate use of GenAI in academic activities.
    1.2.3 To promote the ethical use of GenAI and prevent academic dishonesty.
    1.2.4 To establish a normative framework that guarantees a secure, ethical and responsible use of GenAI.

The development of appropriate guidelines and regulations for the use of AI tools by both academic staff and students in all facets of education—including course creation, teaching, assessments, assignments and grading—has been urged by, among others, Masters [40] and Perera and Lankathilaka [41], who suggest active engagement of relevant stakeholders to ensure the responsible and safe implementation of LLMs [42]. Masters [40] describes three steps for this process, highlighting ethical aspects of AI use: the development of an Education Ethics Committee; the education of the existing Research Ethics Committee; the appointment of a Chief AI Ethics officer. In their review of AI in HE, Perera & Lankathilaka [41] propose specific implementation guidelines based on the following concepts: (1) to raise awareness of the potential uses and limitations of GenAI tools; (2) to use ChatGPT as a complementary tool; (3) to incorporate proctored, in‐person assessments; (4) to develop ethical principles and guidelines; (5) to promote originality and creativity in assignments; (6) to prevent plagiarism; (7) to provide personalised feedback; and (8) to use formative and authentic assessment, such as self‐assessment, reflection reports, portfolios, and peer feedback.

As the literature emphasises, HEIs should be proactive in exploring the impact and utility of GenAI in education and addressing the associated ethical and pedagogical challenges, while promoting its responsible use [4, 13]. In a recent UNESCO global survey of over 450 schools and universities, it was reported that less than 10% have developed institutional policies or formal guidance concerning the use of GenAI applications [43]. The use of ChatGPT and similar LLMs as educational tools requires institutions to carefully consider the academic objectives they aim to achieve [4]: frameworks, guidelines, and policies must address these objectives. Such documents, rather than holding back development, empower universities to implement GenAI‐enabled learning environments and strategies, with a focus on reinforcing rather than replacing human capabilities and decision‐making processes. They should be clear, appropriate and transparent, and enable educators and students to address ethical and privacy concerns in their implementation [4, 7].

Working within such frameworks, HEIs will also need to consider adapting curricula through the introduction of courses covering AI basic concepts (AI literacy), ethical considerations, and practical applications in various disciplines and sciences [23, 42]. These updated curricula will prepare students for professional lives where GenAI will be part of their everyday work [18]. Such a proactive approach enhances educators' and students' GenAI proficiency and literacy, empowers educators to use innovative teaching and learning methods, and improves communication over acceptable and empowering uses of this technology.

2 Human‐Centricity, Inclusion, Non‐discrimination and Fairness

2
**HUMAN‐CENTRICITY, INCLUSION, NON‐DISCRIMINATION AND FAIRNESS**

The GenAI tools used within EUC should be designed or chosen to benefit the educational community, promoting fairness, inclusion, and non‐discrimination. By inclusion, we refer to GenAI tools and interface that provide learning opportunities for different learning styles and needs, are accessible to all learners, including disability relevant accessibility requirements, are fostering adaptive learning and personalised support, as universally designed learning environments. GenAI tools may also be used as tools for removing barriers and provide accessibility for equal opportunities in learning. The outcomes of GenAI tools should always be reviewed with critical judgement and accompanied by the necessary disclaimers to ensure ethical use.

Similar to other internet‐based technologies, GenAI has the possibility to enhance equity and inclusiveness, for example, by improving the writing skills of non‐English speaking individuals [18]. Still, concerns exist that it could increase disparities by supporting privileged students, such as those who can afford updated, paid versions [18].

In their review of institutional policies of universities, McDonald et al. [18] reported that 60% of the universities mentioned in their guidelines the topics of Diversity, Equity and Inclusion.

Examples of good and poor practise in promoting fairness, inclusion, and non‐discrimination were also added as illustrative of these points, as follows:

**Example of Good Practise (Inclusion/Non‐Discrimination)**

**Inclusive Language in Educational Materials**: A best practise for inclusion and non‐discrimination is ensuring that all educational content—whether in assignments, exam questions, or class discussions—is designed using inclusive language. This includes being mindful of diverse gender identities, ethnicities, and backgrounds, and ensuring that materials are accessible to students with disabilities.

*Example*: An instructor creates course content using gender‐neutral language (e.g., ‘they’ instead of ‘he/she’) and ensures that resources are accessible to students with visual impairments by providing screen reader‐friendly formats or subtitles for videos.

**Example of Poor Practise (Inclusion/Non‐Discrimination)**

**Excluding or Marginalising Specific Groups**: A poor practise would involve using biased language or excluding certain groups from learning activities or discussions. This could also include failing to make course materials accessible to all students, particularly those with disabilities.

*Example*: An instructor assigns readings that predominantly feature content from a single gender or ethnic perspective, without providing diverse viewpoints, or neglects to ensure accessibility for students with hearing impairments by not offering captions for video lectures.

3 Respect for Human Rights and Data Privacy

3
**RESPECT FOR HUMAN RIGHTS AND DATA PRIVACY**

  EUC is dedicated to safeguarding the personal data privacy of our students and academic staff. Individuals whose data is used should be informed and give their explicit consent. Clear information and use of standardised forms should be provided to obtain explicit consent, ensuring easy withdrawal options. Consent Management System(s) should be used to track and update consent records and ensure detailed documentation. All contractual clauses with GenAI applications utilised at EUC should be meticulously crafted and reviewed to ensure full compliance with the General Data Protection Regulation (GDPR) and other relevant laws.

If students are to be required to use GenAI, it is essential that data awareness be at the cornerstone of university policy. This is a legal obligation under the European GDPR Act, but it is also essential given the potential threat that data breaches can pose to the safe and secure running of the university. While the threat of data mishandling can pose a threat, conversely it is clear that data awareness and active monitoring of GDPR compliance will create an atmosphere of trust and professionalism. In this context, the framework's insistence on Consent Management Systems is particularly important, as these will mean that all stakeholders will, be constantly involved in data protection efforts. Finally, while at present most use of GenAI in the university is via well‐known major providers which already comply with GDPR, it is likely that future investment in educational technology will require working with more specialised apps, whose compliance will need to be monitored.

4 Safety and Security

4
**SAFETY AND SECURITY**

GenAI tools for teaching and learning at EUC should be adopted only after an assessment of their reliability, transparency and trustworthiness. Where possible, there should be documented monitoring and tracking systems in place to ensure robust and controlled data access and interaction. GenAI tools should be continuously monitored as part of an ongoing proactive cybersecurity strategy. When students are required to use GenAI tools as part of formal assignments in their courses, academic staff is advised to use GenAI tools that have been formally evaluated and approved by the Department of Information Systems at EUC.

Major debates and concerns have animated discussions on the safety and security of GenAI mainly on issues related to data privacy; the potential for biased or unethical outputs; and the risk of AI tools being used maliciously or irresponsibly [44]. Data privacy remains a significant concern as GenAI tools often require access to large datasets, some of which may contain sensitive student information.

When students are required to use GenAI tools as part of formal coursework, it is essential that these tools undergo a formal evaluation process. Specifically, academic staff should prioritise the use of GenAI tools that have been rigorously evaluated and approved by the Department of Management Information Systems at EUC. The department's role in this context is to assess the tools based on criteria such as reliability, data privacy, ethical use, and suitability for academic purposes. This evaluation will ensure that only tools that meet institutional standards are deployed in the academic setting, to maintain consistency, trust, and quality across learning experiences. In addition, when GenAI tools are used from students through the Learning Management Systems used by the HEI, it is essential to establish documented monitoring and tracking systems wherever feasible. These systems will enable academic staff and administrators to closely observe data access, user interactions, and the operational parameters of GenAI tools, thereby ensuring that these interactions remain secure and transparent. Such measures are important in safeguarding both student data and institutional knowledge, promoting a controlled and secure environment where the use of AI enhances rather than compromises academic integrity and privacy.

5 Risks and Opportunities of Generative AI

Debates around GenAI centre on the accuracy, impartiality, impact on critical thinking, teaching methods, interaction quality, plagiarism, academic integrity, and potential to sustain biases and discrimination, all of which that were addressed earlier and also in this part of framework.

5
**RISKS AND OPPORTUNITIES OF GENAI**
  5.1
**Opportunities**
    5.1.1 GenAI tools may enhance creativity by automating routine tasks, allowing students and academic staff to focus on innovative and creative projects. This may lead to new ways of thinking, problem‐solving, and expressing ideas across various disciplines.
    5.1.2 GenAI tools may provide personalised learning experiences by adapting to individual student needs, offering tailored educational content, and providing real‐time, detailed feedback, upon specific and detailed prompting. This can help address diverse learning styles and improve student engagement and outcomes.
    5.1.3 GenAI tools may assist in research by quickly processing and summarising large volumes of information, curating relevant articles, and helping to identify key trends and insights. This can save time and enhance the quality of academic research.
    5.1.4 GenAI tools may provide accessibility requirements for individual learners, including with disabilities by providing e.g., automatic transcription for captioning, speech output and speech recognition, alternative text for visual content, navigation assistance and accessibility testing. This can be of additional support to assistive technology and mainstream technology embedded accessibility.
    5.1.5 GenAI tools may aid in language learning and translation, making educational materials more accessible to non‐native speakers and students with educational needs and disabilities. GenAI‐powered tools may provide translations, transcriptions and assistive technologies that support diverse learning needs.
    5.1.6 In all, GenAI tools may facilitate and support teaching and learning, rather than simply make learning shorter without any tangible learning outcomes. Thus, academic staff is encouraged to:
      - Use GenAI tools in ways that would support the development of learning materials that would enhance student learning, and
      - Develop or revise student assignments based on the features of GenAI‐powered tools, in such ways that the use of GenAI would contribute to the student learning.
  5.2
**Risks**
    5.2.1 GenAI tools can produce false content, rely on inaccurate sources, generate outdated information, and reflect biases present in their training data.
    5.2.2 There are also risks related to copyright issues. GenAI generated content may inadvertently replicate copyrighted materials without proper authorization, leading to potential legal challenges and violations of intellectual property rights. Students and academic staff should be vigilant about the sources of GenAI generated content and ensure proper attribution and permissions are obtained.
    5.2.3 Data privacy concerns are significant when using GenAI tools. Sensitive and personal information provided to GenAI tools may be exposed or misused. It is essential to understand the data handling practises of GenAI tools and to avoid inputting sensitive information unless assurances of data security and privacy are explicitly provided.
    5.2.4 Users of GenAI should recognise their responsibility for the content generated or facilitated by GenAI, ensuring its reliability, accuracy, and relevance while avoiding plagiarism. Awareness of copyright laws and adherence to best practises, including explicit referencing and attention to contractual agreements with suppliers, is essential.
    5.2.5 Overreliance on GenAI may lead to a decline in critical thinking and problem‐solving skills among students. When students depend too heavily on GenAI for generating content and solving problems, they may not develop the necessary analytical abilities and creativity that are crucial for academic and professional success. It is important to use pedagogical approaches that promote student active engagement, critical thinking, creativity and inquiry‐oriented learning with or without GenAI use.

Although most LLM's training data replicates broad general knowledge of many areas through probabilistic pattern recognition, these models become less accurate with more specific queries, lack specialised knowledge (e.g., in healthcare education and practise [45]) and fail to distinguish between evidence‐based and non‐evidence‐based sources. Even the most advanced general models will have difficulty handling ambiguous or highly specialised inputs, which influences the quality and accuracy of their outputs, and thus may negatively influence the intended learning outcomes [2, 7]. Examples of such prompts are inputs that require cultural or contextual knowledge, inputs that require domain‐specific expertise, and inputs that involve complex reasoning, complex language structures, or causal relationships [2]. In a recent study comparing four different LLMs in answering dentistry questions, authors concluded that they demonstrated occasional inaccuracies, errors, outdated or over‐general content and contradictory statements [46]. These limitations hinder the technology's ability to provide comprehensive insights and necessary context, for example in more advanced or complex medical education [2, 42].

To address these deficits, it is suggested that instructors should identify situations where GenAI models might fail to deliver satisfactory responses. In such cases, accurate student prompting and fact‐checking will be vital to guide and correct the chatbot. Creating scenarios that require higher reasoning and understanding than the LLM's training data can help achieve this goal. With such complex questions, it can be beneficial to use assignments and projects that require multiple interactions and feedback from the AI. These promote active learning, which is advantageous for students. Examples of such questions include open‐ended questions, complex problem‐solving, and creative writing prompts. By using AI interactively to tackle these questions, students can enhance their abstract thinking, make connections between various concepts, and understand underlying causal relationships. It is therefore possible that, for instance, the element ‘quality of student‐AI interaction’ might be integrated within a scoring rubric together with other gradable elements of a comprehensive answer or assignment such as clarity, relevance, demonstrated understanding, quality of writing, and originality [2]. In all cases, students must critically assess AI responses and address any ambiguities or limitations through further inquiry and research [2]. Another limitation is that an LLM's static knowledge base has a cut‐off date—for example, at the time of writing ChatGPT had a cut‐off of August 2023, 1 year prior to this paper—meaning it cannot incorporate the latest research, guidelines, and treatment protocols in real‐time. ChatGPT does now have integrated search capabilities, but these are not always reliable guides to best sources. The model might also perpetuate societal biases (e.g., gender, ethnicity, religion and income) because of its training on predominantly English‐language internet sources from high‐income, predominantly white‐dominated Christian or secular countries with colonial histories, leading to potential discrimination [41, 42, 47].

There is also a risk of ‘hallucinations’ producing misleading, nonfactual, inconsistent, or contradictory responses, even when identical prompts are used, whether short or lengthy. This is widely misunderstood as a technical issue that may 1 day be overcome, but such assertions misunderstand the nature of the technology, which is based on probabilistic responses that consider only the likelihood of combinations of words, not their underlying factuality [41, 42]. For this reason, the output of LLMs has been compared to that of parrots which can repeat words without understanding them (the so‐called ‘stochastic parrot’), or, more bluntly but also somewhat more accurately, to Harry G Frankfurt's philosophical definition of ‘bullshit’—words intended to convince the listener or reader of their correctness rather than paying attention to their truth‐value [48, 49, 50]. Teaching students the difference between persuasively factual‐seeming GenAI language outputs and reliable published sources is a key information literacy goal.

Along with these concerns, overreliance on GenAI tools could hinder the development of creativity, critical thinking, problem‐solving, communication, and interpersonal skills [41, 42]. Students might gradually become dependent on AI for answers, even without fully understanding the learning material and the question posed, therefore avoiding engaging with the learning content and neglecting to exercise critical thinking [7]. In addition, the use of LLM chatbots may reduce social interaction between students and educators, thus hindering educational support and decreasing the educational experience [7]. Specifically, in healthcare education, dependence on an AI tool with limited moral judgement capacity might result in future professionals with diminished ethical adherence, trustworthiness, and over‐increased confidence in managing complex cases [42].

Academic integrity and plagiarism are major concerns, as laws and regulations regarding copyright and intellectual property still apply when using content generated by AI. Students may unethically use GenAI content, for example in cases of plagiarism or cheating [7]. To mitigate the potential of academic dishonesty and plagiarism, Shorey et al. [42] propose that assessment methodologies should shift towards evaluating critical and problem‐based thinking competencies. In addition, training data might include copyrighted materials, potentially resulting in content that mimics or duplicates copyrighted works. Adding to this, while ChatGPT has a clear policy that the copyright of generated materials belongs to the user, it is not clear that this is the case in all other current and future GenAI models [51, 52]. Privacy and confidentially could also be compromised, for example, healthcare students and educators risk compromising the confidentiality and security of their personal information and that of their patients when they unknowingly reveal sensitive data through user prompts, particularly since OpenAI openly states that each user's prompt is incorporated into ChatGPT's database to improve the tool's training [42]. These prompts may be used in responses to other users and even included in answers to other users' queries. Moreover, OpenAI reserves the right to share personal information with third parties without needing to notify users or obtain their consent [51].

Aithal and Aithal [3] summarise the threats of ChatGPT on HE as follows: reduced human interaction, dependence on AI, bias and ethical concerns, privacy and security risks, technology dependence and accessibility, pedagogical challenges, legal and ethical considerations. Meanwhile, Masters [40] focuses on ethical aspects and highlights concerns such as data gathering, anonymity, privacy, consent, data ownership, security, bias, transparency, responsibility and autonomy.

On the other hand, GenAI offers important affordances: quick response and ease of use are the most prominent features that capture both students' and educators' interest, together with the simple, friendly and understandable interface [53]. Shorey et al. [42] in their scoping review of ChatGPT's role in healthcare education, identified two main themes: (1) enhancing healthcare education, research and writing, (2) controversies and concerns regarding AI in healthcare education.

ChatGPT and similar LLMs can also be used for problem‐solving, data analysis, clarification of concepts, personalised learning, assessment, language corrections, and editing, and for in‐class and homework assistance [7, 16]. Students using GenAI may reduce their stress, as they can ask for explanations outside the judgmental classroom environment, they can improve their language skills, enhance their self‐confidence and learning experience, develop professional skills, and save time while having immediate answers to questions [54]. Moreover, it is argued that interactions with LLMs while prompting and evaluating outputs, may enhance students' soft skills, such as logical reasoning, problem‐solving, structured and critical thinking, as well as collaboration and emotion regulation [9]. These skills, along with the digital skills, are essential for students to succeed in the digital, interconnected professional world [9]. Faculty can use AI for teaching enhancement, for example to create tests and quizzes, to identify supplementary reading material, relevant examples and explanations, to explain complex concepts and to provide feedback to students. They can also use it for research purposes, for example, data collection and analysis, article summaries, research question generation, and for the improvement of academic writing [16, 41].

In healthcare education, an LLM interface can function as a virtual teaching assistant, offering real‐time feedback, continuous information access, and personalised learning adjustments [54]. This aids in simplifying complex medical topics and supports learners effectively. Additionally, GenAI can reduce faculty workload by streamlining tasks such as syllabus planning, content creation, and assessment drafting, allowing educators to focus on personal and professional development. It also enhances student engagement through dynamic, interactive learning experiences, such as virtual lab simulations, rather than traditional text‐based chatbots [42, 55].

6 Communication and Transparency

A responsible and ethical deployment of GenAI among university instructors and students is of the upmost importance [42]. This section of the EUC Framework refers to the critical aspect of openness to and acceptance of AI tools within the HE environment and to the clarity regarding its possibilities and shortcomings. Instead of banning or penalising, the University chooses to raise awareness of the potential uses of GenAI and to motivate educators to use it for in‐class teaching, along with discussing with students about its advantages and disadvantages [41].

6 **COMMUNICATION AND TRANSPARENCY**
  6.1 Academic staff should provide clear communication to students about the use of GenAI tools in their courses each semester by each instructor. Depending on discipline, this may include issues of transparency about AI's capabilities and limitations, usage examples, citation requirements and consequences for misuse.
  6.2 Classroom discussions may include assessments of student familiarity with GenAI tools, discussions about the ethical implications of GenAI, and reflections on the purpose of writing and originality in academic work.
  6.3 Academic staff should provide guidance on the ethical and responsible use of GenAI, including data protection, intellectual property rights, and information reliability.
  6.4 Overall, academic staff should support academically ethical and responsible use of GenAI by their students, indicating the usefulness of developing skills to appropriately use GenAI but also developing skills specific to the discipline they study.

Examples of good and poor practise in communication were also added as illustrative of these points, as follows:

**Example of Good Practice in Communication**:

An instructor incorporates a discussion on the implications of AI for their discipline in the first week of the course, including teaching their students about the limitations of the technology and the major ethical issues raised by its use. They communicate class policies on the use of AI in writing, both at the start of the course and as part of the instructions for assignments.

**Example of Poor Practice in Communication**:

An instructor vaguely explains that the use of generative AI is not permitted in their class, without clearly defining what counts as proper or improper use, which leads to confusion. In consequence, some students avoid taking advantage of the positive aspects of GenAI, while others use it in improper ways and end up losing marks or failing unnecessarily.

A duty of educators across a wide variety of disciplines is to effectively communicate the strengths and limitations of AI tools to their students, while emphasising the need to critically evaluate the responses and apply cross‐verification techniques to increase the reliability and/or utility of the output [2, 42, 56]. Mc Donald et al. [18] suggest that instructors should talk to students about policies and ethics of AI use, including the learning process and critical thinking, academic integrity, data privacy concerns, dependence on GenAI and implications of human‐AI collaboration.

Educators should also teach students about ‘prompt engineering’, which involves crafting well‐structured and specific queries (prompts) to achieve useful outcomes when interacting with large language models [18, 57]. In the AI context, ‘prompt engineering’ has emerged as a valuable new skill for students [9], currently taught by only a minority of universities [18]. Thoughtfully designed prompts, or series of prompts, help LLMs produce more relevant and accurate responses, and this applies even more for complex scientific queries, such as medical, dental, or other highly specific queries [46]. Providing GenAI with accurate and specific information, and describing the context (intended purpose, audience, language and tone) is essential to obtain the desired output. If the response is unsatisfactory, users can offer detailed feedback to enhance the chatbot's reply and produce a more relevant output [8].

Walter [57] calls the situation of the novice prompter trying to get a GenAI chatbot to create precisely the needed output the ‘prompt‐wise tabula‐rasa problem’, and suggests multiple techniques for getting the best results, including Automatic Prompt Engineer (APE), Generated Knowledge Prompting (GKn), and Tree‐of‐Thought (ToT0 prompting: in most cases, better results will be achieved with an iterative prompting process that refines original results, rather than with a single blunt query).

7 Learning to Use AI

7 **USE OF GENAI BY STUDENTS**
  7.1 **Learning to Use GenAI**
    7.1.1 Institutional support may include the development of structured and/or other organised approaches to GenAI learning, communication of GenAI strategies, and integration of GenAI training sessions into the curriculum using innovative methods such as online learning materials, practical hands‐on sessions, and peer‐learning. Such support is understood to be subject specific and is best developed by collaborating academic staff within Departments and Schools in collaboration, if needed, with the Office of the Vice Rector for Academic Affairs.
    7.1.2 Instructional support may involve explaining the opportunities and limitations of GenAI tools, promoting ethical use, and enhancing critical thinking through structured guidance and practical examples. Academic staff may utilise learning modules developed through course content projects or the Office of the Vice Rector for Academic Affairs that promote student learning for the opportunities and limitations of GenAI tools, promoting ethical use, and enhancing critical thinking.
    7.1.3 Students should be educated on the responsible and efficient use of GenAI tools, with a focus on understanding potential biases, plagiarism, outdated content and the possible lack of transparency in GenAI outputs.
  7.2 **Appropriate Use of AI**
    7.2.1 Students may benefit from being taught to use GenAI tools to enhance their learning and understanding. The use of GenAI should be transparent and ethical, ensuring that GenAI‐generated content is not submitted as original work.
    7.2.2 Students should acknowledge any use of GenAI tools in their assignments and explain how these tools were used, to maintain academic integrity. For instance, students may be requested to shortly state the use of GenAI‐powered tools in assignments and briefly explain how they safeguarded the academically ethical and responsible use of GenAI in their assignments.

This section of the framework was constructed to help both educators and students consider the best ways that GenAI might be employed in student work. The following illustrative examples were appended:

**Example of Good Practice in Student Use of AI:**

A student with an essay assignment uses an LLM as a brainstorming partner, selecting from suggestions made by the chatbot. They then draw up a rough plan and use GenAI to help refine it. They conduct their own research for acceptable sources and write their own essay, drawing on the planning and brainstorming they conducted with the LLM, then use spelling and grammar checkers (another form of AI) to help polish their English. They follow the policy given by their instructor on citing artificial intelligence. Throughout this process, the student has retained control of the intellectual work.

**Example of Poor Practice in Student Use of AI:**

A student copies the essay prompt given by their instructor into a GenAI chatbot, then hands in the response as their own work, with only light editing. In this process, the student has done little to no intellectual labour and therefore has not learned anything from the essay writing process.

The relationship between HE students and GenAI has been extensively researched, starting with focusing on the negative aspects of plagiarism and contract cheating [58] and continuing with broader perspectives focusing on the advantages and learning opportunities offered [59].

Chatbots can, for example, help students improve their writing skills by identifying grammatical and structural issues and providing personalised feedback. They offer tailored guidance based on individual writing styles, highlighting specific areas for improvement [7, 60]. In the future, by analysing student data, such text generators might be able to identify certain student‐specific needs and learning styles, and provide tailored recommendations and support, something that would potentially enhance student engagement [4, 59]. Additionally, language models effectively simulate human conversations, understanding user intent and context to deliver accurate responses. This versatility allows students to explore various fields, including computer programming, essay writing, and solving mathematical problems [7], as well as more creative tasks up to and including writing original high‐quality poetry [56]. When interviewed in the study of Hasanein and Sobaih [7], students stated that ChatGPT was helpful for homework, assignments, and projects, as it provided instant clarifications, explanations, and acted as a virtual tutor offering real‐world examples and addressing specific queries. LLMs can facilitate group activities, enhance peer communication skills, provide immediate assessment, and improve learning efficiency [41, 42, 59]. They were also reported to be suitable to support constructivist learning by allowing students to experiment with ideas, ask questions, and receive instant feedback, helping them build their own understanding and knowledge [7].

As stated in the literature, students should be educated on the proper use and the limitations of GenAI: generation of inaccurate or irrelevant information is possible as explored previously, thus students should cross‐check the produced output [9, 60]. Academic dishonesty is also a concern, as students may write essays without proper citations or allocate the writing to LLMs [59]. Despite these concerns, students can be taught to engage with language models in line with the ethical values of their institutions. First, educators might specify in the course syllabus or assessments that the use of such models is allowed and recommended, explaining its purpose and providing detailed instructions and guidelines for its use [61]. Incorrect uses of generative AI may in some circumstances amount to plagiarism, though the applicability of concepts of plagiarism to GenAI is disputed [62]: nonetheless, part of the instructor's response may include educating students on plagiarism, that is, what plagiarism is, why it is wrong, and its consequences [58]. Educators should guide the students through their interactions with AI to ensure an ethical and productive learning process [61]: students should acknowledge the assistance of the language model in their essay or assignment; they should accompany the final report with an audit rail of prompts and answers, where they would identify the contradictions, the false or outdated information, and the steps they followed to achieve a satisfactory answer; and finally, they should present their work orally to explain their process and ensure understanding of the topics, as recommended by Kavadella et al. [5]. In any case, it is admitted that monitoring the tools students use for their at‐home assignments is not possible and not all students will be truthful [9].

8 Use of AI by Educators

GenAI is a challenge for educators and represents a learning curve for them, which cannot be instantly mastered; instead, it needs a structured, deliberate approach, which, apart from training, could include sharing experiences with colleagues to identify and adopt best practises [60]. This can be challenging in an environment in which there is a widely varying level of faculty expertise with GenAI, from highly experienced users to people who have little or no contact with it. It was therefore essential in this part of our Framework to identify the major challenges for educators and give some advice as to the most useful approaches.

8 **USE OF GENAI BY ACADEMIC STAFF**
  8.1 **GenAI in Educational Materials**
    8.1.1 Academic staff are encouraged to familiarise themselves with developments in GenAI tools to support their teaching processes. This includes developing course materials, structure and content, interactive activities and assignments, and providing feedback while ensuring that GenAI is used to augment, not replace, their professional judgement, providing an enhanced learning experience for their students.
    8.1.2 EUC shall provide support through training and guidelines to ensure that academic staff adopt GenAI responsibly and effectively.
    8.1.3 Academic staff will benefit from staying informed about GenAI developments and join relevant communities for ongoing engagement.
  8.2 **Interactive and Active In‐Class Teaching for conventional courses**
    8.2.1 Academic staff may integrate GenAI into in‐class teaching to facilitate interactive and active learning. This, among others, may involve using GenAI to present case studies, encourage student engagement, and support of collaborative learning activities based on the course learning outcomes.
    8.2.2 AI tools may be used to generate complex questions that encourage critical thinking and problem‐solving among students.
  8.3 **Interactive and active asynchronous learning and student engagement for e‐learning courses**
    8.3.1 Academic staff may use GenAI to create engaging asynchronous e‐learning activities, such as chatbots, discussions, quizzes and GenAI‐assisted document summaries.
    8.3.2 GenAI can be used to provide timely and personalised feedback on non‐graded assignments, ensuring that students receive the support they need even in an asynchronous learning environment. This can include automated detailed feedback and suggestions for refinements and further study.
    8.3.3 Academic staff may utilise GenAI to facilitate peer collaboration and interaction in asynchronous courses. This can include GenAI‐moderated discussion boards, virtual study groups, and collaborative projects where GenAI helps organise and monitor student participation and progress.
  8.4 **Student Assignments and Grading**
    8.4.1 The use of GenAI for providing feedback to student work should be carefully monitored by the instructor to prevent misuse. GenAI‐powered tools in some disciplines may be able to provide detailed, individualised feedback to students that may enhance their learning trajectories. Academic staff may use GenAI to provide feedback for student assignments while ensuring that the final judgement remains with the instructor. In doing so, academic staff should develop skills for prompt engineering to be able to create meaningful, useful and supportive student learning experiences.
    8.4.2 Students should be informed if GenAI‐powered tools are being used for feedback generation and it should be explained how this feedback was used, monitored and approved by the instructor.
  8.5 **Preventing Misuse and Academic Dishonesty**
    8.5.1 Measures should be implemented to prevent the misuse of GenAI tools and uphold academic integrity. Such measures include promoting student awareness about the ethical use of GenAI and monitoring assignments for potential misuse.
    8.5.2 Plagiarism detection tools, while useful, have limitations and may be misused. Academic staff should be aware of the ethical implications of such tools, be prepared to mitigate biased outputs, and explore alternative ways to address academic integrity concerns. Such work should be done collaboratively within frameworks established by individual departments, recognising the subject‐specific nature of these issues.
    8.5.3 In cases of suspected misuse of GenAI, academic staff should prefer their own expertise over GenAI detection tools and should handle the issue by following EUC regulations on academic ethics and students' discipline. Meanwhile, academic staff should make sure that they develop student assignments in such ways that:
      - They consider that the students will make some use of GenAI‐powered tools for the completion of the assignments, and
      - GenAI may be used as a tool that facilitates teaching and learning, and not facilitate academic cheating.

The following examples of best and less good practises were appended:

**Example of Good Practise (grading and feedback):**

An instructor grades an essay using a rubric, adding observations in bullet point form where useful. These notes form observations are then run through GenAI to produce detailed, personalised, and fully explained feedback.

**Example of Poor Practise (grading and feedback):**

The instructor uploads the student's assignment directly to a third‐party site and copies/pastes the feedback received without checking it. This runs the risk of breaking data protection laws, and the AI cannot assess the assignment in the context of the specific class so will grade inaccurately.

**Example of Good Practise (assignments):**

In class, an instructor asks students to discuss what AI tools they use for their studies and uses the discussion as an opportunity to provide specific advice regarding issues of data protection, intellectual property rights, information reliability, and transparency.

**Example of Poor Practise (assignments):**

An instructor presents examples of proper AI technologies and their possible benefits when used in homework assignments, without, however, explaining the pitfalls and misuses that the students should avoid.

GenAI can be a valuable tool for instructors in HE. For example, ChatGPT can create custom exercises and quizzes, offer feedback, and generate tailored educational materials aligned with a student's learning style and progress. In addition, ChatGPT can assist in developing ideas for lectures, drafting seminar plans and course descriptions, and crafting announcement texts [9]. Innovative assessment formats are also possible (and possibly necessary) while using GenAI tools. Examples include oral presentations where students who have performed assignments using GenAI will have the opportunity to demonstrate their understanding of the learning material, collaborative projects promoting teamwork, reflective activities on a completed task, and even alternative formats such as videos and animations [9]. In their analysis of institutional guidelines and policies, Mc Donald et al. [18] reported that only one‐third of institutions provided guidance for faculty on using GenAI for lesson planning, while more than half (63%) provided guidance on designing assignments discouraging the use of GenAI by students.

In the study of Godsk and Elving [63], faculty responses to using ChatGPT for academic purposes in HE were mixed. They appreciated ChatGPT's benefits as an always available resource that could be used by students to generate examples and get an overview understanding of most topics, but concerns were also raised about overreliance on the tool, potentially undermining critical thinking. Despite these concerns, the academics recognised ChatGPT's value in enhancing teaching, optimising test preparation, and providing insights to improve student support and teaching strategies.

Correspondingly, academic staff should be trained to increase their literacy around AI, with the aim to be able to evaluate AI tools and use them efficiently and on an informed basis. Schwendicke et al. [23] proposed a core curriculum for dental healthcare educators and learners (applicable in other domains, as well), including four learning outcomes:

(1) Basic definitions and terms, the reasoning behind AI, and the training and validation of models; (2) Use cases, the required types of AI to address them, and the setup of AI software for dental purposes; (3) Evaluation metrics and interpretation, the impact of AI on patient/societal health outcomes and associated examples; (4) Issues around generalizability and representativeness, explicability, autonomy and accountability and the need for governance.

Faculty awareness of AI is crucial, thus faculty should consider how to instil ethical values in students in relation to academic and scientific conduct, while training them in academic integrity and the responsible use of GenAI language models [7]. In healthcare education particularly, ethical considerations related to patient privacy and data security must be explicitly communicated to students [3]. Instructors may also consider advising students on ways to use GenAI to support—not replace—primary learning, along with urging them to verify ChatGPT's information and recognise that while prompt engineering can yield more unique answers, AI models have limitations and their outputs should always be critically evaluated [3]. A comprehensive education might involve consulting diverse sources, such as textbooks, academic journals, and experts, and incorporating critical thinking into the teaching and learning process.

AI utilisation by instructors might include developing students' critical thinking and problem‐solving skills [7]. To that end, instructors might develop complex questions that require multiple feedback and interactions with AI to obtain an answer [2]. For example, open‐ended questions that oblige the student to engage in conversation with the AI, or problem‐solving questions that require the student to navigate several stages of reasoning and analysis to reach a solution [2]. An experimental study has demonstrated that leveraging ChatGPT during didactic assistance in‐class activities can positively impact students' critical, creative and reflective thinking skills [64].

In view of the advent of GenAI, a redefinition of the role of educators may occur, involving focusing more on advanced teaching areas while delegating routine tasks like grading and answering common questions to LLM‐enabled chatbots. This shift allows educators to concentrate on curriculum development, research, mentorship and fostering critical thinking [4, 65]. However, over‐reliance on ChatGPT could potentially lower educational standards and raise privacy concerns due to the extensive student data it requires [4], so in the end, academic staff must balance the use of ChatGPT with human expertise. While ChatGPT is a valuable supplementary tool, it is meant to complement, not replace, the knowledge and judgement of educators. Thus, human instructors are essential for providing and ensuring effective learning experiences [42].

9 AI And GDPR Compliance

9 **Data Privacy regarding AI**

The following principles will guide the use of Generative AI in teaching and learning at EUC to ensure compliance with GDPR:

9.1.1 **Lawful Basis for Data Processing**

Using Generative AI for teaching and learning should always comply with all requirements concerning the legal basis they rely on. For example, if they rely on consent, explicit consent will be obtained from individuals before their personal data is used in Generative AI applications. The consent process will clearly explain how the data will be used, the purposes of its use, and the individuals' rights.

9.1.2 **Lawfulness, Fairness and Transparency**

Personal data must be processed lawfully, fairly, and transparently. It must be ensured that the use of Generative AI in educational settings is communicated clearly to all stakeholders, staff, students and data subjects are informed about how their data will be used.

9.1.3 **Purpose Limitation**

Personal data will be collected for specified, explicit and legitimate purposes and not further processed in a manner that is incompatible with those purposes. Generative AI applications will only use data for educational purposes explicitly stated at the time of data collection.

9.1.4 **Data Minimization**

Only data that is necessary for the purposes of teaching and learning will be collected and processed. Efforts will be made to minimise the amount of personal data used by Generative AI applications.

9.1.5 **Anonymization and Pseudonymization**

The GDPR emphasises using anonymization and pseudonymization techniques to safeguard personal data and enhance an individual's privacy. The GDPR does not regard anonymized data as personal data. Pseudonymization lowers the risk of re‐identifying personal data. However, pseudonymized data is still considered personal data. Anonymization and pseudonymization are essential techniques in relation to the operation of AI systems that process personal data.

9.1.6 **Accuracy**

All reasonable steps must be taken to ensure that personal data is accurate and, where necessary, kept up to date. Inaccurate data will be rectified or deleted without delay.

9.1.7 **Storage Limitation**

Personal data will be kept in a form that permits identification of data subjects for no longer than is necessary for the purposes for which the personal data is processed.

9.1.8 **Integrity and Confidentiality**

Personal data will be processed in a manner that ensures appropriate security, including protection against unauthorised or unlawful processing, accidental loss, destruction or damage. Appropriate technical and organisational measures must be implemented to secure data used in Generative AI applications.

9.1.9 **No Automated Decision‐Making**

EUC confirms that no automated decision‐making, including profiling, will take place using Generative AI technologies within the educational environment. All decisions impacting students and staff will be made by qualified individuals to ensure fairness, transparency, and accountability.

9.1.10 **Data Subject Rights**

All the rights of data subjects must be respected, including the right to access their data, the right to rectification, the right to erasure, the right to restrict processing, and the right to data portability. Procedures will be established to facilitate these rights.

9.1.11 **Third‐Party Processors**

Any third‐party processors involved in the implementation of Generative AI will be thoroughly vetted to ensure they comply with GDPR standards. Data processing agreements will be in place to enforce compliance.

9.1.12 **Cross‐Border Data Transfers**

GDPR emphasises ensuring appropriate safeguards for cross‐border transfers of personal data. Therefore, transfers of any personal data internationally must have sufficient controls in place, such as Standard Contractual Clauses (SCCs).

9.1.13 **Accountability**

EUC will be responsible for, and be able to demonstrate, compliance with these principles. Records of data processing activities related to Generative AI will be maintained.

The following examples of best and less good practices were appended:

**Examples of Good Practice (GDPR):**

**For Students Using ChatGPT:**

**Data Minimization**: Students should avoid entering personal or sensitive information when using ChatGPT. For instance, students should refrain from sharing private data such as their full name, student ID, or health information in conversations with the model. This aligns with the GDPR principle of data minimization, ensuring that only necessary and relevant data is processed.

*Example*: A student asking for help with a general programming question without providing their personal contact details or assignment‐specific information.

**For Instructors Using ChatGPT:**

**Anonymization of Student Data**: Instructors should ensure that any data shared with ChatGPT or any other AI tool for educational purposes is anonymized. This means stripping out personal identifiers like names, grades, or specific coursework details before processing any data. This minimises the risk of personal data being inadvertently shared and ensures GDPR compliance.

*Example*: When seeking assistance for grading rubrics or creating general course content, an instructor should avoid mentioning students' names or sharing any identifiable information in queries.

**Examples of Poor Practise (GDPR)**

**For Students Using ChatGPT:**

**Sharing Personal Data Without Consent**: A poor practise would be for students to share personal, identifiable information such as their full name, address, or login credentials with ChatGPT, which violates the GDPR principle of consent and privacy.

*Example*: A student providing their full name, email address, and personal assignment details when asking for help with specific homework.

**For Instructors Using ChatGPT:**

**Processing Students' Sensitive Data Without Proper Safeguards**: Instructors could be at risk of breaching GDPR by uploading students' personal data (e.g., grades, personal feedback) into an AI tool like ChatGPT without appropriate safeguards or consent. Sharing such information could lead to GDPR violations, especially if the tool processes data in ways that students are unaware of.

*Example*: An instructor discussing specific student performance or personal academic histories in queries to ChatGPT without anonymizing the data.

This section addresses critical data protection principles to ensure compliance with the GDPR, emphasising various GDPR principles, such as lawful data processing, transparency, purpose limitation, data minimization, anonymization, and pseudonymization. These principles are aligned with concerns raised in the literature regarding the risks associated with data privacy and ethical data handling in AI applications [66, 67]. Reported concerns include the improper use of personal data, the lack of transparency in data collection processes, and the risks of re‐identification of anonymized data, which could lead to breaches of privacy [68].

The framework incorporates measures to ensure data accuracy and integrity, stipulating that personal data must be up‐to‐date and secure. This aligns with the literature's emphasis on the need to prevent the misuse of inaccurate data, which could lead to faulty AI‐driven decisions [69]. Additionally, the prohibition of automated decision‐making reflects concerns about fairness and accountability in AI, ensuring that decisions affecting students and staff are made by qualified individuals rather than relying solely on potentially biased algorithms [67].

The principles related to third‐party processors and cross‐border data transfers further address concerns about the involvement of external entities in data processing. Third parties should comply with GDPR standards and having sufficient controls for international data transfers are crucial for safeguarding data privacy and security. Finally, the accountability principle reinforces the need for institutions to not only comply with data protection standards but also to demonstrate such compliance through documented processes.

10 Conclusion

This section, finally, was added to stress the fast‐moving nature of advances in AI, introducing a further note of flexibility and emphasising personal responsibility.

10
**CONCLUSION**

**Continuous Improvement**

Given the dynamic nature of GenAI, it is crucial to stay informed about updates, new tools, and evolving best practises. Continuous engagement with the latest developments will ensure effective navigation of the challenges and opportunities presented by AI tools.

The implementation of LLMs is an iterative process driven by evaluation and feedback for continuous improvement. Inputs like student and teacher feedback, classroom observations, and performance data help identify shortcomings and ensure relevance. As a result, guidelines should be regularly updated to maintain legitimacy and educational quality [70]. The emphasis on continuous change towards the end of the EUC framework points to the ever more fast‐moving environment in which educators in all disciplines find themselves. On the one hand, there are evident possibilities in a technology that can automate repetitive administrative tasks and assist students in practicing their skills and refining their knowledge. Machine learning, of which generative AI is just one example, also has the potential to enhance research, in dentistry and many other disciplines. On the other hand, given that the automated tools developed to detect GenAI‐written text are not fully reliable, and that many of them can frequently be evaded with only minimal editing of outputs, much of the initial discussion has naturally focused on the possibilities of student cheating, resulting in many educators developing negative attitudes. Additional caution has been raised as faculty have become more familiar with issues around the veracity and trustworthiness of GenAI outputs, which it should be stressed are a feature of a probabilistic text generator and will likely always exist within this form of the technology. Concerns over the effect on students' learning journeys of their outsourcing any part of the thinking process are also reasons to be careful regarding its implementation. As AI evolves, LLMs may become more personalised, interactive and integrated into teaching and learning. Ethical, privacy, and pedagogical concerns will require continuous discussion and research, as will the long‐term effects of LLM usage on students' learning experiences and well‐being, the impact on teaching practises and potential implications for educational equity [70].

It is in this environment of rapid change and uncertainty over benefits and drawbacks that a framework like the EUC document is essential. As readers will have observed, the document was written in order to allow maximum flexibility. Indeed, such flexibility was a major part of the process. Once we had come up with our major principles, we instructed ChatGPT 4o to rewrite them in a style that was internally consistent and also consistent with previous documents issued by the university. In doing so, the GenAI made many of the points in the document rigidly proscriptive, with most of them starting with some variant of ‘educators/students **should**…’ During the human revision process, which involved several editors, many of these were changed to ensure that faculty control over their own research and teaching was emphasised, using words that stressed agency. (The exception, of course, being rules to do with academic honesty). This is the advantage of a flexible framework such as this document over more rigid rulebooks or even guidelines. Frameworks can be used to stress principles rather than prescriptions, the resulting plasticity being the best way to ensure that faculty and students actively engage with these new methods of text and image production rather than passively relying on instructions.

**FIGURE 2:**
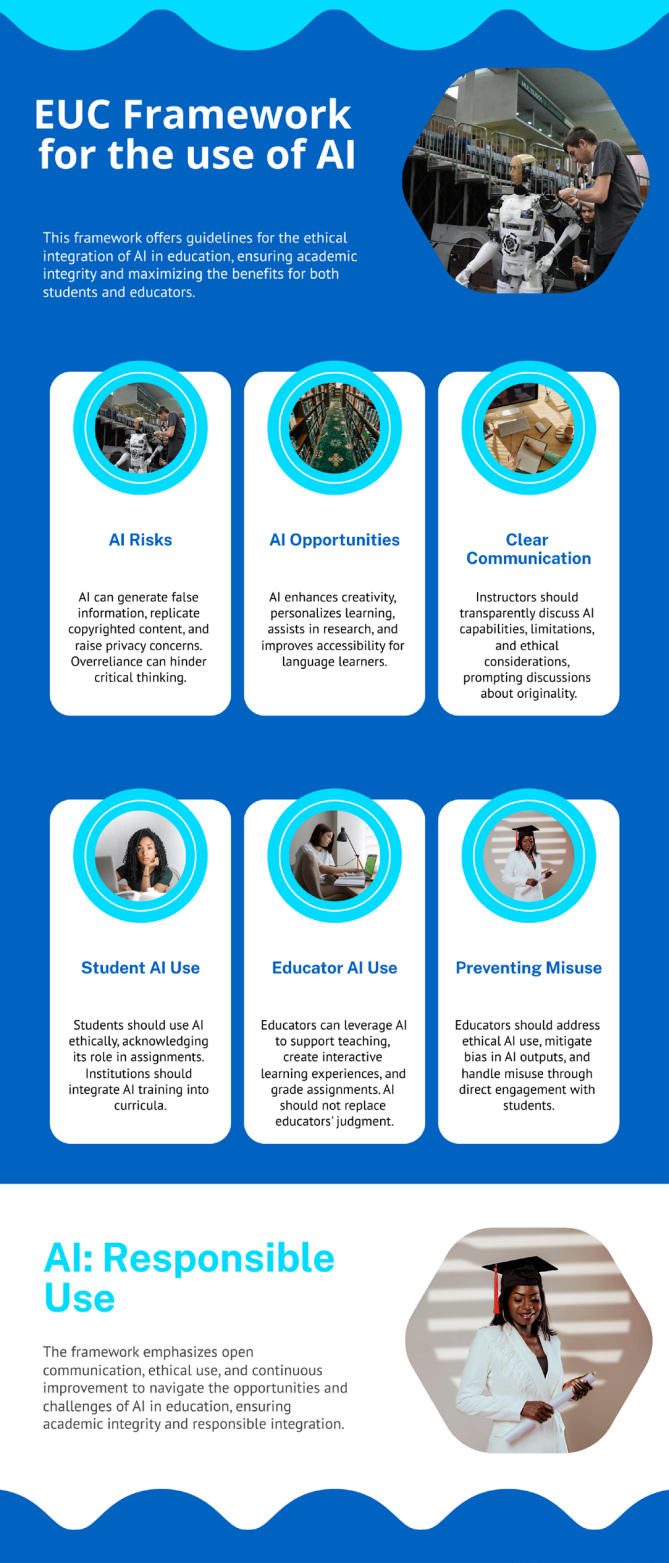
Infographic.

To summarise the guidance framework, an Infographic was developed (using Piktochart, an AI‐powered infographic generator) for the dissemination of the AI Framework, through the University's social media, webpage, newsletters, etc. (Figure 2). Early feedback on our work on integrating the technology by establishing frameworks of principles has been positive, and we will continue to work to improve our institution's readiness for changes and developments still to come, building on our own research and the work of others. We have shared our process and the resulting document in the hope that it will be of use to others, particularly those in academic leadership roles.

As mentioned before, a UNESCO survey of over 450 educational institutions found that as few as 10% had formal guidance in place for educators and students regarding GenAI [43]. This may reflect uncertainty over the current place of such technologies on a discipline‐by‐discipline basis, or fears of future developments making any rules obsolescent. We recommend that all universities should embark on a process of consultation and develop similarly flexible frameworks to help faculty and students incorporate GenAI in their current practise, and we intend to release our own framework on a Creative Commons licence so that other HEIs can make use of it, either centrally or on a departmental basis, adapting it to their unique needs.

## Conflicts of Interest

The authors declare no conflicts of interest.

## Data Availability

The authors have nothing to report.
